# Type V aplasia cutis congenita

**DOI:** 10.4103/0256-4947.60530

**Published:** 2010

**Authors:** Umar A. Qureshi, Nisar Ahmed

**Affiliations:** a From the Department of Neonatology and Pediatrics, Sher-i-Kashmir Institute of Medical Sciences, Srinagar, Kashimr, India; b From the Department of Radiodiagnosis, Sher-i-Kashmir Institute of Medical Sciences, Srinagar, Kashimr, India

**Figure 1:**
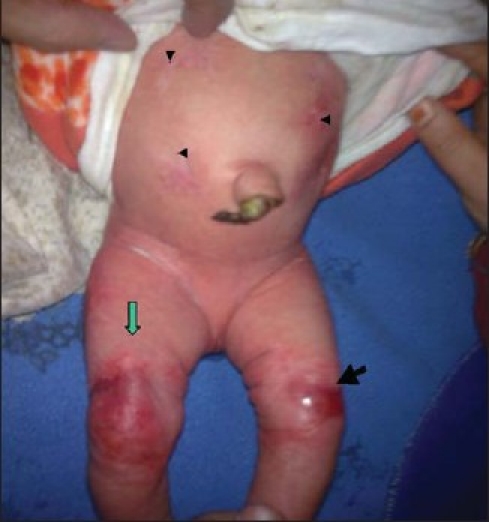
Translucent blister (green arrow), well demarcated superficial skin erosion (black arrow), and initial stages of scar formation (arrowheads).

**Figure 2:**
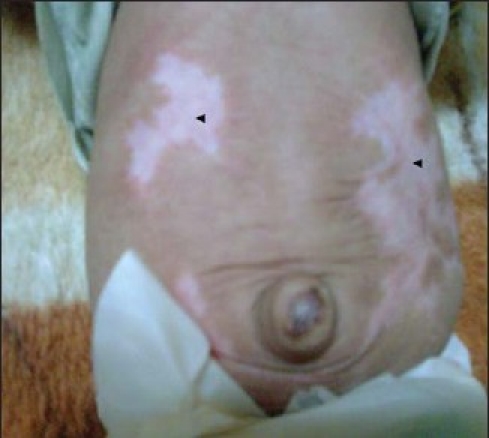
Atrophic pale fibrous scars (arrowheads).

A two-hour-old female neonate, a product of nonconsanguineous marriage with unremarkable family history, was brought with well-defined bilaterally symmetrical and superficial erosions on the knees, trunk and lower limbs with sparing of scalp and mucosa (Figure 1). Biopsy revealed an absence of epidermis and superficial dermis. Antenatal records revealed that the mother was diagnosed with a twin pregnancy at 12 weeks gestation with a follow-up scan revealing pregnancy loss (fetus papyraceous). There was no maternal history of drug intake. There were no other congenital malformations. Ultrasonography of the head and abdomen and karyotyping and screening for intrauterine infections were normal. The child was managed conservatively with paraffin gauze dressings. The lesions evolved into fibrous scars (Figure 2). No new skin lesions appeared. A diagnosis of type V aplasia cutis congenita (ACC) with fetus papyraceous was made.

Type V ACC is a nonscalp cutis aplasia with fetus papyraceous.^1^ It results from temporary hypotension in the surviving twin at the time of fetal demise in the early second trimester leading to poor perfusion and skin necrosis.^2^ The lesions of type V ACC are typically bilaterally symmetrical. The lesions are noninflammatory, well demarcated and vary in size from 0.5 to 10 cm. Diagnosis is based on the physical appearance of the skin and biopsy. The appearance of the lesions and histological features at birth vary depending on when they occur during intrauterine development and depth of aplasia. Lesions that form early in gestation may heal before delivery and appear as fibrotic scars, whereas less mature defects may present as an ulceration of variable depths (erosions/ulcers). ACC must be differentiated from the epidermolysis bullosa (mucosal involvement), Transient bullous dermolysis of the newborn resolves during the first months of life leaving no dystrophic scars. Treatment includes gentle cleansing and applying bland ointment or silver sulfadiazine ointment.
